# Drug-resistant Nontyphoidal *Salmonella* Bacteremia, Thailand^1^

**DOI:** 10.3201/eid1303.061059

**Published:** 2007-03

**Authors:** Wanla Kulwichit, Tanittha Chatsuwan, Chudaachhara Unhasuta, Chaiwat Pulsrikarn, Aroon Bangtrakulnonth, Anan Chongthaleong

**Affiliations:** * Chulalongkorn University, Bangkok, Thailand; and †World Health Organization National *Salmonella* and *Shigella* Center, Bangkok, Thailand

**Keywords:** Drug-resistant Nontyphoidal *Salmonella* Bacteremia, Thailand, Nontyphoidal Salmonella, drug resistance, quinolones, cephalosporin, nalidixic acid, ceftriaxone, ciprofloxacin, bacteremia, bloodstream infections, food animals, letter

**To the Editor:** Despite improved public health, serious infections with nontyphoidal *Salmonella enterica* remain a major clinical and public health concern in Thailand and worldwide (*1*,*2*). Life-threatening *Salmonella* infections resistant to fluoroquinolones, extended-spectrum cephalosporins, or both, have been increasingly reported (*3*). Use of antimicrobial drugs for disease prevention and growth promotion in food animals has been implicated in this increase in drug resistance (*4*). Because of extensive global travel, such increases affect the medical community domestically and internationally (*5*). We report a pilot survey of drug resistance in *Salmonella* spp. in Thailand.

We studied archival nontyphoidal *Salmonella* isolates from bacteremic patients at King Chulalongkorn Memorial Hospital from January 2003 to October 2005 and from bacteremic patients in Thailand sent to the World Health Organization National *Salmonella* and *Shigella* Center in Bangkok during the first half of 2005. The isolates from these archives were nonoverlapping and were kept frozen at −80°C. Isolates were divided into *Salmonella* serovar Choleraesuis and other nontyphoidal *Salmonella* (non-Choleraesuis) because we observed that Choleraesuis isolates show a higher frequency of resistance to fluoroquinolones and extended-spectrum cephalosporins than non-Choleraesuis isolates. A standard Etest method (AB Biodisk, Solna, Sweden) was used to evaluate MICs for nalidixic acid, ciprofloxacin, and ceftriaxone. Susceptibility was defined according to the 2005 criteria for *Salmonella* of the Clinical Laboratory Standards Institute (CLSI, formerly NCCLS) (*6*).

Isolates showed high frequencies of antimicrobial drug resistance (Figure). All *S*. Choleraesuis isolates with ceftriaxone resistance also showed high levels of resistance to nalidixic acid (MIC ≥256 µg/mL); most of these also had reduced susceptibility to ciprofloxacin (MIC ≥0.125 µg/mL). Of 73 nalidixic acid–resistant *Salmonella* isolates, 55 (75%) required a ciprofloxacin MIC ≥0.125 µg/mL, 14 (19%) required an MIC of 0.094 µg/mL, and 4 (6%) required an MIC of 0.064 µg/mL. One patient with aortitis caused by ceftriaxone-resistant *S.* Choleraesuis died of a ruptured mycotic aneurysm.

**Figure:**
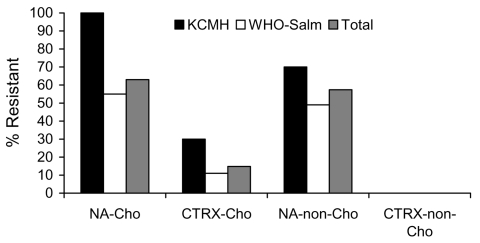
Percentage of nontyphoidal *Salmonella* isolates resistant to nalidixic acid (NA) and ceftriaxone (CTRX), Thailand. KCMH, King Chulalongkorn Memorial Hospital; WHO-Salm, World Health Organization *Salmonella* and *Shigella* Center. Cho, Choleraesuis; non-Cho, non-Choleraesuis. The analysis included 10 Cho isolates from KCMH, 44 Cho isolates from WHO-Salm, 27 non-Cho isolates from KCMH, and 41 non-Cho isolates from WHO-Salm. Two Cho isolates from WHO-SAlm with intermediate MICs for ceftriaxone are also included.

In the food animal industry, the effect of using antimicrobial drugs has long been a subject of concern (*7*–*9*). Evidence from molecular epidemiologic studies (*9*) suggests that these concerns are genuine and that serious problems must be addressed. This concern is also supported by reports of fatal, invasive, nontyphoidal *Salmonella* infections resistant to quinolones or extended-spectrum cephalosporins (*7**,**10*). In Thailand, enrofloxacin, a veterinary fluoroquinolone, is used in animals in the poultry, swine, and seafood industries. Ceftiofur, a third-generation cephalosporin, is used extensively in swine for treatment and prevention of disease and for growth promotion. When compared with previous susceptibility patterns (*5*), current nontyphoidal *Salmonella* infections in humans in Thailand are more resistant to quinolones and cephalosporins. Susceptibility to nalidixic acid correlates well with reduced susceptibility to ciprofloxacin. An alarming increase in ceftriaxone resistance in *S*. Choleraesuis may be associated with inappropriate cephalosporin use in swine farming. Major revisions in current policies for use of antimicrobial drugs in food animals in Thailand are warranted.
